# Linc00963 Promote Cell Proliferation and Tumor Growth in Castration-Resistant Prostate Cancer by Modulating miR-655/TRIM24 Axis

**DOI:** 10.3389/fonc.2021.636965

**Published:** 2021-02-11

**Authors:** Minghua Bai, Chenchen He, Shengjia Shi, Mincong Wang, Jinlu Ma, Pengtao Yang, Yiping Dong, Xingyi Mou, Suxia Han

**Affiliations:** ^1^ Department of Radiation Oncology, The First Affiliated Hospital of Xi’an Jiaotong University, Xi’an, China; ^2^ Department of Radiation Oncology, The Second Affiliated Hospital of Xi’an Jiaotong University, Xi’an, China; ^3^ Department of Andrology, Assisted Reproductive Technology Center, Northwest Women’s and Children’s Hospital Affiliated to Xi’an Jiaotong University, Xi’an, China; ^4^ Department of Clinical Medicine, Xi’an Jiaotong University Health Science Center, Xi’an, China

**Keywords:** Linc00963, TRIM24, miR-655, proliferation, CRPC

## Abstract

Previous studies have shown that both long intergenic non-coding RNA 00963 (Linc00963) and tripartite motif containing 24 (TRIM24) are activators of the PI3K/AKT pathway, and both are involved in the carcinogenesis and progression of prostate cancer. However, the regulatory mechanisms between Linc00963 and TRIM24 are still unclear. In this study, we aimed to elucidate the underlying relationship between Linc00963 and TRIM24 in castration-resistant prostate cancer (CRPC). We found that TRIM24, an established oncogene in CRPC, was positively correlated with Linc00963 in prostate cancer tissues. In addition, TRIM24 was positively regulated by Lin00963 in CRPC cells. Mechanistically, TRIM24 was the direct target of microRNA-655 (miR-655) in CRPC cells, and Linc00963 could competitively bind miR-655 and upregulate TRIM24 expression. Using gain- and loss-of- function assays and rescue assays, we identified that miR-655 inhibits TRIM24 expression and cell proliferation and colony forming ability in CRPC, and that Linc00963 promotes TRIM24 expression, cell proliferation, and colony forming ability of CRPC cells by directly suppressing miR-655 expression. We further identified that Linc00963 could promote tumor growth of CRPC cells by inhibiting miR-655 and upregulating TRIM24 axis *in vivo*. Taken together, our study reveals a new mechanism for the Linc00963/miR-655/TRIM24 competing endogenous RNA (ceRNA) network in accelerating cell proliferation in CRPC *in vitro* and *in vivo*, and suggests that Linc00963 could be considered a novel therapeutic target for CRPC.

## Introduction

A total of 191,930 estimated diagnosed prostate cancer cases and 33,330 estimated prostate cancer related deaths are expected in 2020 in the United States, accompanied by a drastically increased incidence and mortality in China in the last decade (1, 2). Despite continuous improvements in diagnosis and treatment, the currently recommended maintenance schedules from androgen deprivation therapy (ADT) to radical resection are only effective in patients with hormone-sensitive prostate cancer (HSPC). However, HSPC deteriorates to castration-resistant prostate cancer (CRPC), and the five years overall survival rate remains extremely disappointing (3). Previous studies have confirmed that the phosphatidylinositol 3-kinase/serine/threonine kinase (PI3K/AKT) signaling pathway has pivotal functions in the carcinogenesis and developmental process of prostate cancer (4), especially in the transition from HSPC to CRPC. PI3K/AKT inhibitors also showed excellent therapeutic effects in preclinical studies and phase I clinical trials of CRPC (5). Thus, it is important to elucidate the regulation mechanism of the PI3K/AKT pathway in CRPC progression.

Numerous evidences have confirmed that long non-coding RNAs (LncRNA) play a pivotal role in CRPC by functioning as oncogenes or tumor suppressors (6, 7). Previously, we generated differentially expressed lncRNA profiles of LNCaP and C4-2 cells and proved that long intergenic non-coding RNA 00963 (Linc00963) is abnormally upregulated in CRPC cells, and found that silencing Linc00963 expression in C4-2 cells attenuated cell proliferation, migration, and invasion ability *in vitro*, and inhibited epidermal growth factor receptor (EGFR) and phosphatidylinositol-4,5-bisphosphate 3-kinase, catalytic subunit alpha (PIK3CA) expression which have synergistic roles in PI3K/AKT pathway activation (8). Recently study found Linc00963 could function as a ceRNA to upregulate nucleolar protein homolog 2 (NOP2) expression and promote cancer metastasis by sponging microRNA (miR)-542-3p in prostate cancer (9). However, the underlying mechanism underlying Linc00963 mediated enhanced proliferation in CRPC was still not elucidated.

The expression levels of tripartite motif-containing protein 24 (TRIM24) were also elevated from HSPC to CRPC, and could enhance the abilities of cell proliferation and PIK3CA and EGFR expression in CRPC cells (10). Importantly, we found silencing the expression of TRIM24 could suppress cell proliferation abilities and invasion-metastasis cascade in CRPC *in vitro* and *in vivo* (11). Notably, TRIM24 was positively correlated with cancer development and chemo-resistance in prostate cancer and glioma by activating the PI3K/AKT pathway (10, 12). However, it is unclear whether there are regulatory mechanisms between Linc00963 and TRIM24, which are both PI3K/AKT pathway activators. Therefore, we examined the relationship between TRIM24 and Linc00963 to uncover the mechanisms underlying Linc00963-mediated enhanced proliferation in CRPC *in vitro* and *in vivo* in the current study.

## Materials and Methods

### Cell Culture

LNCaP, PC-3, and C4-2 human prostate cancer cell lines, and RWPE1, a human prostate epithelial cell line, were purchased from GeneChem (Shanghai, China). Keratinocyte serum free medium (K-SFM, Gibco, NY, USA) containing calf pituitary extract and EGF was used to culture RWPE1 cells, and Dulbecco’s modified eagle medium (DMEM, Gibco) containing 10% fetal bovine serum (FBS, Cellmax, Beijing, China) and 1% penicillin-streptomycin (Cellmax) was used to culture LNCaP, C4-2, and PC-3 cells. All cells were cultured at 37°C in a humidified atmosphere with 5% CO_2_.

### Construction of Lentivirus Expression Vector

Lentiviral-Linc00963-wild type(Lv-Linc00963-WT or Linc00963)/mutant (Linc00963/MUT) and negative control lentivirus (Lv-control) were designed as described previously (13), and were obtained from Genechem (Shanghai, China). In brief, the full length human Linc00963 with WT or MUT miR-655 binding sites and negative control were cloned in to Age I and Bam I sites of the CV146 core vector. Then, Lipofectamine 2000 was used to transfect 20 μg CV146-Linc00963-WT/MUT/NC, 15 μg pHelper 1.0, and 10 μg pHelper 2.0 into HEK293T cells. The medium was changed to 10% DMEM after 8 h and the cell supernatant was collected after 72 h, followed by centrifugation at 4°C for the concentration and purification of Lv-Linc00963 and Lv-control.

### Lentivirus Infection and siRNA/miRNA Transfection

Lentivirus infection and siRNA/miRNA transfection were performed as described previously (14, 15). Briefly, for lentivirus infection, HiTransG A (Genechem) was used to facilitate infection of Lv-Linc00963/NC into PC-3 or C4-2 cells. Then, medium containing puromycin (Concentration: 2 μg/L) was used to selected PC-3 and C4-2 cells for two weeks in order to obtain stable Linc00963-upregulated cells. The stable Linc00963-upregulated cells were then collected for WB, RT-QPCR, CCK-8, EdU assays, and colony forming assays. TRIM24 siRNA, scrambled NC siRNA, miR-655 mimics, miR-655 inhibitors, and miR-655 NC were synthesized and provided by Ribo Bio (Guangzhou, China). For siRNA/miRNA transfection, Lipofectamine 2000 (ThermoFisher, USA) was used to transfect the siRNA (100 nM)/miRNA (50 nM) into PC-3 and C4-2 cells. Transfected cells were then harvested for RT-QPCR, CCK-8, WB, EdU assays, and colony forming assays 48 h later. The lentiviral and siRNA sequences are shown in **Table 1**.

**Table 1:** Sequence of lentivirus and siRNAs used in the experiments.

Name	Sequence
Lv-Linc00963 sense	5’- AGGTCGACTCTAGAGGATCCCCGGCCCGTCTCGGGGCCCTGAGTC-3’
Lv-Linc00963 antisense	5’-CACAGGCTAGCTCAACCGGTTTATGCTGAAAATATTCCAAGGTTTATTG-3’
Lv-control sense	5’-AGGGTACCCCTGGGACCGGTCGCCACCTAGGCGGGACCGGAGACG-3’
Lv-control antisense	5’-TCCACTAGGTGCGGACCGGGCGTGAACTCCCAATGAGCA-3’
TRIM24 siRNA sense	5’-GCCACCAAGUGGUUUAUCATT-3’
TRIM24 siRNA anti sense	5’-UGAUAAACCACUUGGUGGCTT-3’
NC siRNA sense	5’-UUCUCCGAACGUGUCAGGUTT-3’
NC siRNA antisense	5’-AGGUGACACGUUCGGAGAATT-3’

### Cell Count Kit-8, Colony Forming Assays, and Western Blot

The proliferation of different transfected PC-3 and C4-2 cells was measured using CCK-8 assays. The colony forming ability of different transfected groups was measured by the plate colony forming assay. Protein levels of TRIM24 in different transfected PC-3 and C4-2 cells were measured by WB. All the detailed procedures for plate colony forming, CCK-8, and WB were conducted as described previously (14).

### EdU Staining

Proliferation of different transfected PC-3 and C4-2 cells was further evaluated using the Cell-Light EdU Apollo567 *in vitro* Kit (Ribo Bio). Briefly, 10^5^ cells seeded in 96-well plates, were stained with 100 μl 50μM EdU solution for 2 h in the dark at room temperature. Then, 4% paraformaldehyde was used to fix the cells for 30 min, and 0.5% Triton X-100 was used to permeabilize the cells for 15 min. Finally, the cells were stained with Apollo^®^567 and DAPI. Representative images were taken using the confocal microscope (Olympus, Japan) at ×200 magnification.

### RNA Pull-Down Assay

RNA pull-down assays were conducted as described previously with a few modifications (13). Briefly, NP40 lysis buffer was used to lyse PC-3 cells, and 1 mg cell extracts were incubated with a biotin-labelled Linc00963-probe or Linc00963-MUT-probe at 4°C for 6 h. Subsequently, RNAs with biotin-labelled NC (Bio-NC-probe), Linc00963 (Bio-Linc00963-probe) or Linc00963-MUT (Bio-Linc00963-MUT-probe) were mixed with 40 μl streptavidin agarose beads and incubated overnight on a rotator. Finally, the expression of miR-655 in the retrieved RNA was identified using RT-QPCR as we described in Results 2.9.

### Luciferase Assay

Luciferase assays were performed as described in our previous study, with a few modifications (16). pmirGLO-wild type (WT)-Linc00963/TRIM24 vector was constructed by cloning the 3′-untranslated region (UTR) of Linc00963 or TRIM24 containing miR-655-binding sites into pMirGLO dual-luciferase miRNA target expression vector (Promega Corporation, WI, USA). To construct the pmirGLO-mutant (MUT)-Linc00963/TRIM24, the QuikChange™ site-directed mutagenesis kit (Stratagene; now owned by Agilent Technologies, Inc., Santa Clara, CA, USA) was used to introduce mutations from U to C into the potential miR-655 binding sites of Linc00963. The dual-luciferase reporter assay system (Promega Corporation) was used to measure the relative firefly luciferase activities. *Renilla* luciferase activity served as an internal control.

### Fluorescence In Situ Hybridization

Oligonucleotide probes for Linc00963 and U6 were purchased from Ribo Bio (Guangzhou China). PC-3 cells were seeded in 20-mm confocal dishes. After overnight incubation, cells were fixed with 4% paraformaldehyde for 20 min, and permeabilized with 0.3% Triton X-100 for 90 s. Then, hybridization buffer containing Linc00963 and the U6 FISH probe was incubated with the cells overnight at 37°C, in dark. Then, DAPI was used to stain the nucleus on the next day. A confocal microscope (Olympus) was used to acquire images at ×400 magnification.

### Quantitative Real-Time Polymerase Chain Reaction

The protocols of RNA extraction and RT-QPCR were described in our previous study (17). The primers were obtained from Sangon Biotech (Shanghai, China) and their sequences are shown in **Table 2**.

**Table 2:** Primers used in the experiments.

Name	Sequence
Linc00963 forward (human)	5’- AGGAGCAACAGCGAAGGT -3’
Linc00963 reverse (mouse)	5’- TCTGTGGTGCGTGTCTGC -3’
β-actin forward	5’- GGCGGCACCACCATGTACCCT -3’
β-actin reverse	5’- AGGGGCCGGACTCGTCATACT -3’
TRIM24 forward	5’-GCAGGTGAAGAAGGCTCGAT-3’
TRIM24 reverse	5’- CCCAGAATGATGAGCAAGCA-3’
miR-655 forward	5’-AATAGTGCCTAAAGTGCTGC-3’
miR-655 reverse	5’-AGACCCACCTCAATCATCCT-3’
U6 forward	5’-CTCGCTTCGGCAGCACA-3’
U6 reverse	5’-AACGCTTCACGAATTTGCGT-3’

### Animal Experiments

All animal experiment procedures followed the guidelines of the Guide for Animal Care and Use Committee of Xi’an Jiaotong University. Each group had five Balb/c athymic nude mice (nu/nu; weight: 16–18 g; age: 6 weeks). In total, 5 ×10^6^ PC-3 cells with Linc00963 stable upregulation or control cells were subcutaneously injected into the left flank of each mouse. After the tumors were visible, tumor length and width were measured every 3 days for 2 weeks. Tumor volumes were calculated using the formula: 0.5 × length × width^2^. After two weeks, the mice were sacrificed and the tumors were collected and weighed. Tumor specimen sections were stained with Ki-67 antibody after deparaffinizing and rehydration.

### Statistical Analysis

Statistical analysis was performed by using IBM SPSS statistical software (version 22.0). Student’s t-test was used for data analysis and *P* values were determined using 2-sided tests. Statistical significance was considered when *P* < 0.05.

## Results

### Linc00963 Is Positively Associated With TRIM24 in Tissues and Cells of Prostate Cancer

**Figure 1:**
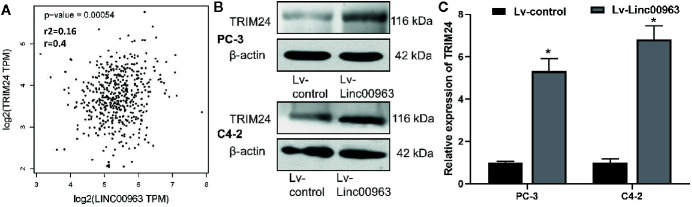
TRIM24 is positive correlated with Linc00963 in castration-resistant prostate cancer (CRPC) tissues and cells. **(A)** Correlation of Linc00963 and TRIM24 expression in 492 prostate adenocarcinoma (PRAD) patients from The Cancer Genome Atlas (TCGA) database, which was analyzed by using the Gene Expression Profiling Interactive Analysis website (http://gepia.cancer-pku.cn/); **(B)** The effects of Linc00963 on TRIM24 protein levels in CRPC cells; **(C)** The effects of Linc00963 on TRIM24 mRNA levels in CRPC cells; Data are shown as the mean ± S.E. *P < 0.05. The representative results of 3 independent experiments are shown.

Correlation of Linc00963 and TRIM24 expression in the tissues of 492 prostate adenocarcinoma (PRAD) patients from The Cancer Genome Atlas (TCGA) database, which was analyzed by using the Gene Expression Profiling Interactive Analysis website (GEPIA, http://gepia.cancer-pku.cn/), we found the relative expression of Linc00963 was positively correlated with the relatively levels of TRIM24 mRNA (*P*<0.05, **Figure 1A**). Furthermore, both protein and mRNA levels of TRIM24 were upregulated in PC-3 and C4-2 cells transfected with Lv-Linc00963 compared to those in cells transfected with Lv-control (*P* < 0.05, the effects of upregulation of Linc00963 on TRIM24 protein levels are shown in **Figure 1B**, the effects of upregulation of Linc00963 on TRIM24 mRNA levels are shown in **Figure 1C**). These results indicate that Linc00963 positively correlated with TRIM24 expression in the tissues and cells of prostate cancer.

### Linc00963 Directly Binds miR-655 in CRPC Cells

**Figure 2:**
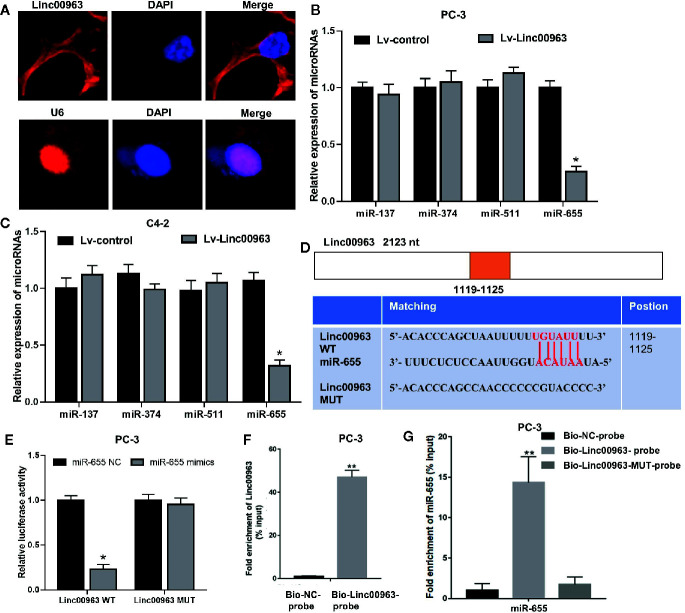
Linc00963 directly sponges miR-655 in castration-resistant prostate cancer (CRPC) cells. **(A)** Subcellular localization of Linc00963 in PC-3 cells, detected by FISH; Blue: DAPI, Green: Linc00963-probe; **(B, C)** Effects of Linc00963 on the expression of miRNAs with putative binding sites for TRIM24 in PC-3 **(B)** and C4-2 **(C)** cells; **(D)** wild type (WT) and mutant (MUT) targeting region of Linc00963 for miR-655; **(E)** Relative luciferase activity in PC-3 cells co-transfected with WT/MUT Linc00963 plasmid and miR-655 NC/mimics; **(F)** Enrichment of Linc00963 in the sample pulled down by the Bio-Linc00963-probe and Bio- NC-probe; **(G)** Enrichment of miR-655 in the sample pulled down by the Bio-Linc00963-probe, Bio-Linc00963-MUT-probe, and Bio- NC-probe; Data are shown as the mean ± S.E. *P < 0.05, **P < 0.01. The representative results of 3 independent experiments are shown.

To further elucidate the underlying mechanism of Linc00963 and TRIM24 in CRPC, subcellular localization of Linc00963 was confirmed by FISH. As shown in **Figure 2A**, Linc00963 was localized in both the nuclei and cytoplasm (**Figure 2A**), suggesting that Linc00963 might either bind with RNA-binding proteins or function as a ceRNA in CRPC cells. A recently study identified Linc00963 could function as a ceRNA to upregulate NOP2 expression and promote cancer metastasis by sponging miR-542-3p in prostate cancer (9). Thus, we explored whether Linc00963 could also act as ceRNA to enhance TRIM24 expression and further promote cell proliferation in CRPC. TRIM24 has been identified as a direct target of miR-137, miR-374, miR-511, and miR-655 in prostate cancer (18) and gastric cancer (19). However, only miR-655 was found to be significantly downregulated following the enhancement of Linc00963 expression in PC-3 and C4-2 cells (P < 0.05, **Figures 2B, C**). The levels of miR-137, miR-374, and miR-511 were not changed following Linc00963 upregulation in CRPC cells (P > 0.05, **Figures 2B, C**). Thus, we selected miR-655 as the potential downstream target of Linc00963 in CRPC. In line with our hypothesis, we found that miR-655 did contain the putative binding sites for Linc00963 by searching online bioinformatics databases (miRDB: http://mirdb.org/). Then, Linc00963 WT and MUT vector were constructed to identify the relationship between Linc00963 and miR-655 in CRPC cells (**Figure 2D**). Co-transfecting Linc00963 WT vector and miR-655 mimics into PC-3 cells resulted in a significant decline in the relative luciferase activity (P < 0.05, **Figure 2E**). In contrast, the relative luciferase activities were not significantly changed in PC-3 cells co-transfected with Linc00963 MUT vector and miR-655 mimics (P > 0.05, **Figure 2E**). More importantly, RNA pull-down assays demonstrated that miR-655 could be pulled down by the Bio-Linc00963-probe (P < 0.01. **Figures 2F, G**), but not the Bio-Linc00963-MUT-probe in PC-3 cells (P > 0.05, **Figure 2G**). Thus, we concluded that Linc00963 could directly sponge miR-655 in CRPC cells.

### miR-655 Inhibits Cell Proliferation and Colony Forming Ability in Castration-Resistant Prostate Cancer

**Figure 3:**
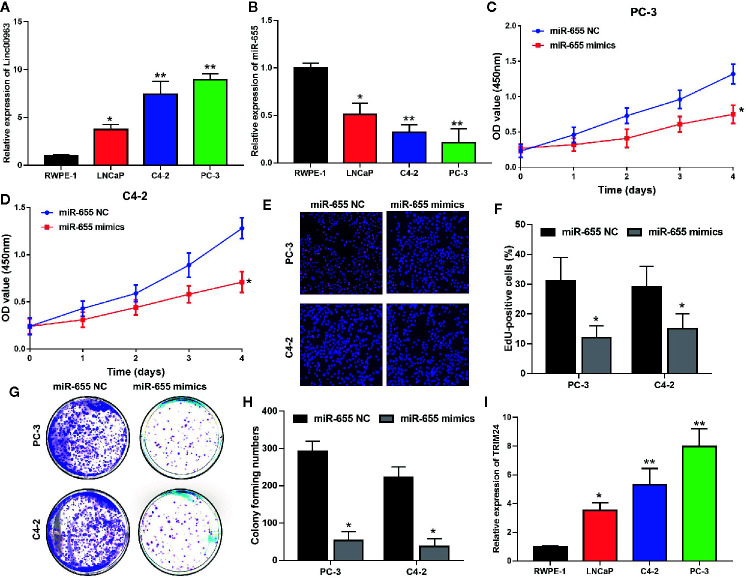
miR-655 inhibits cell proliferation and colony formation of castration-resistant prostate cancer (CRPC) cells. **(A)** The expression of Linc00963 in RWPE-1 and prostate cell lines, detected by quantitative real-time polymerase chain reaction (RT-QPCR); **(B)** The expression of miR-655 in RWPE-1 and prostate cell lines, detected by RT-QPCR; **(C, D).** The effects of miR-655 on cell proliferation of PC-3 after transfection of 50 nM miR-655 mimics for 48 h **(C)** and C4-2 **(D)** cells, detected by CCK-8; **(E)** The effects of miR-655 on cell proliferation after transfection of 50 nM miR-655 mimics for 48 h, detected by EdU assay; **(F)** Statistical analysis of EdU positive cells in CRPC cells transfected with miR-655 NC or mimics; **(G)** The effects of miR-655 on colony formation of CRPC cells after transfection of 50 nM miR-655 mimics for 48 h; **(H)** Statistical analysis of colony forming ability in CRPC cells transfected with miR-655 NC or mimics; **(I)** TRIM24 expression in the normal epithelial cell line RWPE-1 and in prostate cell lines, detected by RT-QPCR; Data are shown as the mean ± S.E. *P < 0.05, **P < 0.01. Representative results of 3 independent experiments are shown.

Consistent with our previous study, Linc00963 expression was higher in prostate cancer cells (LNCaP, PC-3, C4-2) than that in RWPE-1 (*P*< 0.05, **Figure 3A**). Importantly, the expression of Linc00963 was further enhanced in CRPC cells (PC-3, C4-2) compared with that in primary prostate cancer cells (LNCaP) (*P* < 0.05, **Figure 3A**). In contrast, the levels of miR-655 were lower in prostate cancer cells (LNCaP, PC-3, C4-2) than those in RWPE-1 cells (*P* < 0.05, **Figure 3B**). More importantly, miR-655 expression was further decreased in PC-3 and C4-2 cells compared with that in LNCaP (*P* < 0.05, **Figure 3B**). The proliferation rates of PC-3 and C4-2 transfected with miR-655 mimics were inhibited compared to those in cells transfected with miR-655 NC (*P* < 0.05, **Figures 3C–F**, the effects on proliferation detected by CCK-8 are shown in **Figures 3C, D**, the effects on cell proliferation detected by Edu are shown in **Figures 3E, F**). Moreover, cell colony forming ability in PC-3 and C4-2 cells transfected with miR-655 mimics was decreased compared with that in cells transfected with miR-655 NC (*P*< 0.05, **Figures 3G, H**). Thus, we conclude that miR-655 inhibits cell proliferation and is a potential tumor suppressor in CRPC.

### TRIM24 Is the Downstream Target of miR-655 in Castration-Resistant Prostate Cancer

**Figure 4:**
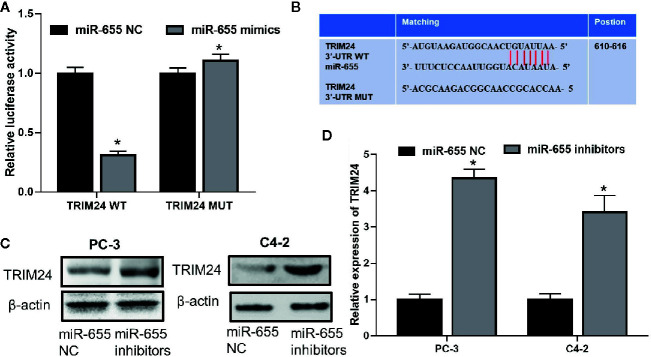
TRIM24 is the direct target of miR-655 in castration-resistant prostate cancer (CRPC) cells. **(A)** Relative luciferase activity in PC-3 cells co-transfected with wild type (WT)/mutant (MUT) TRIM24 plasmid and miR-655 NC/mimics; **(B)** Putative binding sequence of miR-655 in the 3′-UTR of TRIM24; **(C)** Effects of miR-655 on TRIM24 protein levels in PC-3 and C4-2 cells after transfection of 50 nM miR-655 inhibitors for 48 h, detected by western blot (WB); **(D)** Effects of miR-655 on TRIM24 mRNA levels in PC-3 and C4-2 cells after transfection of 50 nM miR-655 mimics for 48 h, detected by RT-QPCR; Data are shown as the mean ± S.E. *P < 0.05.

We further investigated whether there was a regulatory mechanism between miR-655 and TRIM24 in prostate cancer. TRIM24 mRNA levels were upregulated in prostate cancer cells (LNCaP, PC-3, C4-2) compared with those in RWPE-1 (*P*< 0.05, **Figure 3I**); these were inversely associated with miR-655 expression and positively associated with Linc00963 expression in prostate cancer. More importantly, co-transfecting TRIM24 3′-UTR WT vector and miR-655 mimics into PC-3 cells resulted in a significantly declined relative luciferase activity (*P*< 0.05, **Figures 4A, B**). Whereas, the relative luciferase activities in PC-3 cells co-transfected with TRIM24 3′-UTR MUT vector and miR-655 mimics were not significantly changed (*P*> 0.05, **Figure 4A**). Furthermore, decreased miR-655 levels induced enhanced expression of TRIM24 at protein (**Figure 4C**) and mRNA (**Figure 4D**) levels in PC-3 and C4-2 cells.

### miR-655 is One of the Functional Mediators of Linc00963 in Castration-Resistant Prostate Cancer

**Figure 5:**
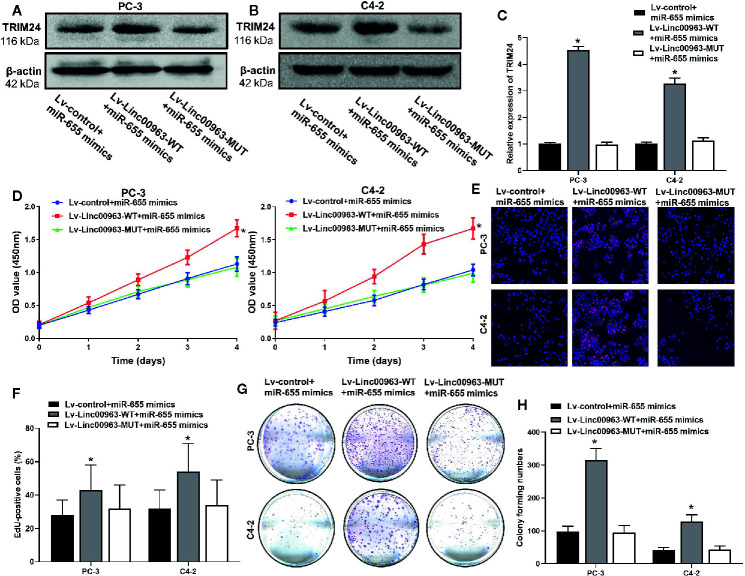
miR-655 is one of the functional mediator of Linc00963 in castration-resistant prostate cancer (CRPC) cells. **(A, B)** The effects of Linc00963 with or without binding sites of miR-655 on TRIM24 protein levels in miR-655 mimics transfected CRPC cells; **(C)** The effects of Linc00963 with or without binding sites of miR-655 on TRIM24 mRNA levels in miR-655 mimics (concentration: 50 nM; time: 48 h) infected CRPC cells; **(D)** The effects of Linc00963 with or without binding sites of miR-655 on cell proliferation in miR-655 mimics transfected CRPC cells, detected by CCK-8 assay; **(E)** The effects of Linc00963 with or without binding sites of miR-655 on cell proliferation in miR-655 mimics (concentration: 50 nM; time: 48 h) transfected CRPC cells, detected by EdU assay; **(F)** Statistical analysis of EdU-positive cells transfected with Lv-control+ miR-655 mimics, Lv-Linc00963-WT+ miR-655 mimics, and Lv-Linc00963-MUT+miR-655 mimics; **(G)** The effects of Linc00963 with or without binding sites of miR-655 on colony formation in miR-655 mimics transfected CRPC cells; **(H)** Statistical analysis of colony forming numbers in cells transfected with Lv-control+ miR-655 mimics, Lv-Linc00963-WT+ miR-655 mimics, and Lv-Linc00963-MUT+miR-655 mimics; Data are shown as the mean ± S.E. *P < 0.05. Representative results of 3 independent experiments are shown.

Then, we performed rescue experiments to further identify whether Linc00963 promoted proliferation in CRPC though sponging miR-655 expression. WB and RT-QPCR analysis demonstrated that both protein and mRNA levels of TRIM24 were upregulated in PC-3 and C4-2 cells transfected with Lv-Linc00963-WT+miR-655 mimics compared to those in cells transfected with Lv-control+miR-655 mimics, and the effects of Lv-Linc00963 on TRIM24 expression were attenuated in Lv-Linc00963-MUT+miR-655 mimics group (*P*< 0.05, the effects on TRIM24 protein levels are shown in **Figures 5A, B**, the effects on TRIM24 mRNA levels are shown in **Figure 5C**). CCK-8 (*P*< 0.05, **Figure 5D**), EdU (*P*< 0.05, **Figures 5E, F**), colony forming assays (*P*< 0.05, **Figures 5G, H**) demonstrated that enhanced cell proliferation and colony forming ability induced by Linc00963 upregulation in CRPC cells would be rescued when Linc00963 failed to bind with miR-655.

### Linc00963/miR-655 Axis Promote Cell Proliferation Through Enhancing TRIM24 Expression

**Figure 6:**
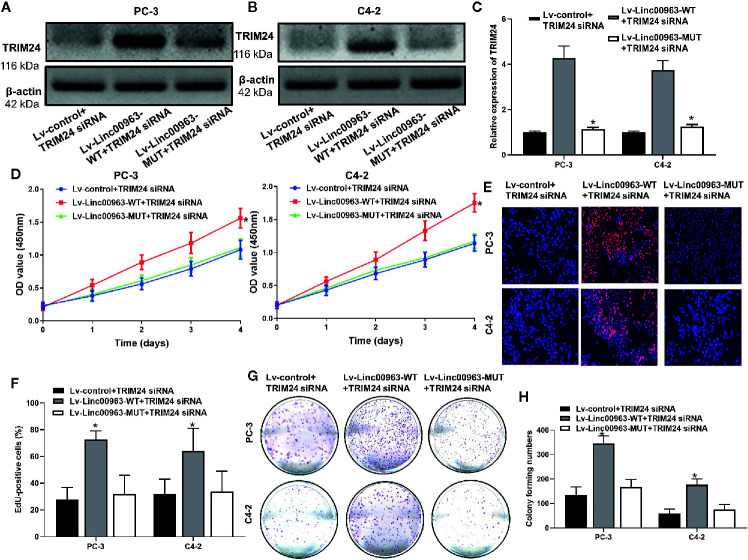
TRIM24 is involved in Linc00963/miR-655 axis-mediated castration-resistant prostate cancer (CRPC) cell proliferation. **(A, B)** The effects of Linc00963 with or without binding sites of miR-655 on TRIM24 protein levels in TRIM24 siRNA (concentration: 100 nM; time: 48 h) transfected CRPC cells; **(C)** The effects of Linc00963 with or without binding sites of miR-655 on TRIM24 mRNA levels in TRIM24 siRNA (concentration: 100 nM; time: 48 h) infected CRPC cells; **(D)** The effects of Linc00963 with or without binding sites of miR-655 on cell proliferation in TRIM24 siRNA (concentration: 100 nM; time: 48 h) transfected CRPC cells, detected by CCK-8 assay; **(E)** The effects of Linc00963 with or without binding sites of miR-655 on cell proliferation in TRIM24 siRNA (concentration: 100 nM; time: 48 h) transfected CRPC cells, detected by EdU assay; **(F)** Statistical analysis of EdU-positive CRPC cells transfected with Lv-control+TRIM24 siRNA, Lv-Linc00963-WT+TRIM24 siRNA, and Lv-Linc00963-MUT+TRIM24 siRNA; **(G)** The effects of Linc00963 with or without binding sites of miR-655 on colony formation in TRIM24 siRNA (concentration: 100 nM; time: 48 h) transfected CRPC cells; **(H)** Statistical analysis of colony forming numbers in CRPC cells transfected with Lv-control+TRIM24 siRNA, Lv-Linc00963-WT+TRIM24 siRNA, and Lv-Linc00963-MUT+TRIM24 siRNA; Data are shown as the mean ± S.E. *P < 0.05. The representative results of 3 independent experiments are shown.

We then investigated whether Linc0063/miR-655 axis promote cell proliferation in CRPC through enhancing TRIM24 expression. We found both protein and mRNA levels of TRIM24 were upregulated in PC-3 and C4-2 cells transfected with Lv-Linc00963-WT+TRIM24 inhibitors compared to those in cells transfected with Lv-control+TRIM24 inhibitors, and the effects of Linc00963 on TRIM24 expression were attenuated in Lv-Linc00963-MUT+TRIM24 inhibitors (*P*<0.05, the effects on TRIM24 protein levels are shown in **Figures 6A, B**, the effects on TRIM24 mRNA levels are shown in **Figure 6C**). In functional terms, CCK-8, EdU assay, and plate colony forming assays indicated that the cell proliferation and colony forming ability were enhanced after upregulation of Linc00963 in TRIM24 inhibitors transfected PC-3 and C4-2cells (*P*<0.05, the effects on proliferation detected by CCK-8 are shown in **Figure 6D**, the effects on cell proliferation detected by EdU are shown in **Figures 6E, F**, whereas those of colony forming ability are shown in **Figures 6G, H**). However, the cell proliferation and colony forming ability of PC-3 and C4-2 cells were partly decreased in Linc00963 MUT+TRIM24 inhibitors group (*P*<0.05, **Figures 6D–H**). These results indicate that Linc00963/miR-655 axis promotes cell proliferation, and colony forming ability by upregulating TRIM24 expression in CRPC.

### Linc00963 Promotes Proliferation of CRPC Cells by Targeting miR-655/TRIM24 Axis *In Vivo*

**Figure 7:**
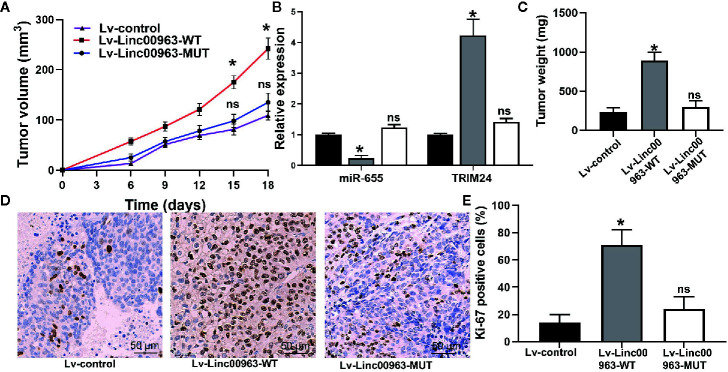
Linc00963 promotes tumor growth of castration-resistant prostate cancer (CRPC) cells. **(A)** Tumor growth curve of PC-3 cells infected with Lv-control, Lv-Linc00963-WT and Lv-Linc00963-MUT; **(B)** Tumor weight of PC-3 cells infected with Lv-control, Lv-Linc00963-WT and Lv-Linc00963-MUT; **(C)** Relative expression of miR-655 and TRIM24 in resected tumor tissues of Lv-control, Lv-Linc00963-WT and Lv-Linc00963-MUT group; **(D)** Ki-67-positive cells in nude mice bearing PC-3 cells infected with Lv-control, Lv-Linc00963-WT and Lv-Linc00963-MUT; **(E)** Statistical analysis of the percentage of Ki-67 positive cells in nude mice bearing PC-3 cells infected with Lv-control, Lv-Linc00963-WT and Lv-Linc00963-MUT; Data are shown as the mean ± S.E. *P < 0.05, ns, not significant. Representative results of 3 independent experiments are shown.

After injecting PC-3 cells into mice, faster tumor growth and bigger tumor volume were observed in the Lv-Linc00963-WT group (*P*<0.05, **Figure 7A**). Consistently, the tumor weight of Lv-Linc00963-WT group was greater than that in the Lv-control, but the tumor weight of Lv-Linc00963-MUT group was similar as the Lv-control group (Lv-control *vs* Lv-Linc00963-WT, *P*<0.05; Lv-control *vs* Lv-Linc00963-MUT, *P*>0.05, **Figure 7B**). Furthermore, the relative expression of TRIM24 was higher and miR-655 was lower in the resected tumor tissues of Lv-Linc00963-WT group than Lv-control group (*P<*0.05, **Figure 7C**). Whereas, the relative expression of TRIM24 and miR-655 were not changed significantly in the resected tumor tissues of Lv-Linc00963-MUT group and Lv-control group (*P*>0.05, **Figure 7C**). More importantly, compared to the Lv-Linc00963-control group, Ki-67 staining also showed more positive cells in the Lv-Linc00963-WT group (*P*<0.05, **Figures 7D, E**). These results indicate Linc00963 functions as ceRNA to promote tumor growth of CRPC cells by targeting miR-655/TRIM24 axis *in vivo*.

## Discussion

**Figure 8:**
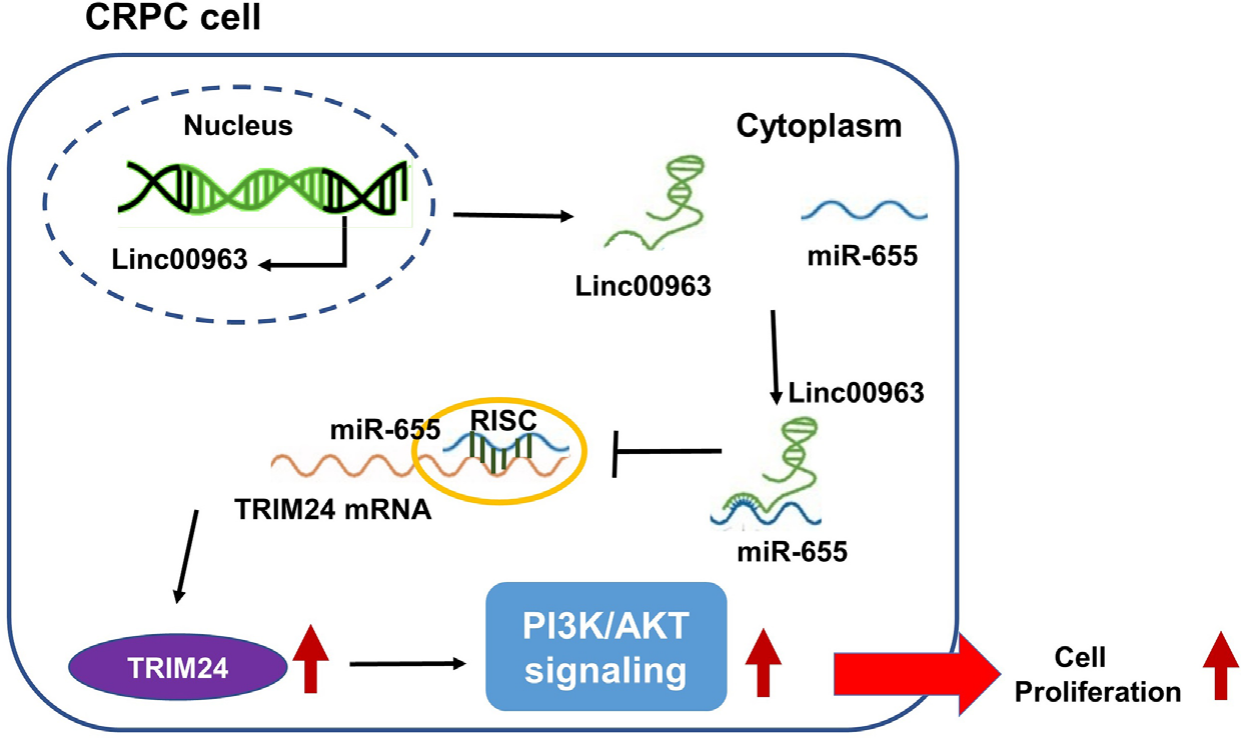
Linc00963 functions as competing endogenous RNA (ceRNA) to enhance TRIM24 expression and promote cell proliferation in castration-resistant prostate cancer by sponging miR-655.

LncRNAs have been identified as prognostic predictors and crucial regulators in multiple cancers, including prostate cancer. For example, LncRNA LBCS suppresses castration resistance and proliferation of prostate cancer by functioning as a scaffold for hnRNPK protein and AR mRNA to inhibit AR translation efficiency (20). LncRNA HOXD-AS1 promotes chemoresistance and cell proliferation of CRPC cells by binding WDR5 protein to mediate H3K4me3 modification in target genes (21). Previously, we found that Linc00963 was upregulated in C4-2 compared to LNCaP by generating comparative lncRNA profiles of the CRPC cell line C4-2 and HSPC cell line LNCaP, indicating that Linc00963 is involved in the transition from HSPC to CRPC (8). Furthermore, we found that silencing Linc00963 expression in C4-2 cells attenuated their proliferation, migration, and invasion ability, and inhibited EGFR and PIK3CA expression, and the phosphorylation levels of AKT, indicating that Linc00963 is a potential oncogenic LncRNA and PI3K/AKT pathway activator in CRPC cells (8). However, we failed to expound the mechanism of Linc00963 in the regulation of cell proliferation and activation of the PI3K/AKT pathway in our previous study (8). Interestingly, a newly published article also found Linc00963 was up-regulated in hepatocellular carcinoma and activated PI3K/AKT signaling pathway (22). In the following study, Linc00963 was proved to be a ceRNA in prostate cancer, which further identified the pivotal role of Linc00963 in the metastasis of prostate cancer (9). Here, we further confirmed that Linc00963 also functioned as a ceRNA to promote cell proliferation in CRPC by targeting the miR-655/TRIM24 axis (**Figure 8**). Both our previous study and the current study identified the oncogenic role of Linc00963 in CRPC.

Numerous evidences indicate that TRIM24 upregulation is significantly associated with cancer development and poor prognosis in multiple cancer types such as bladder cancer (23), gastric cancer (24), non-small cell lung cancer (25), and breast cancer (26). TRIM24 was also identified as an oncogene in advanced CRPC. Groner et al. found that TRIM24 expression was significantly higher in CRPC than primary prostate cancer, and that it was positively correlated with disease recurrence (10). Moreover, they showed that enhanced TRIM24 expression could promote proliferation of CRPC cells by activating AR and the PI3K/AKT pathway at extremely low androgen levels. In our previous study, we confirmed the oncogenic role of TRIM24 in CRPC. We found that targeted silencing of TRIM24 expression resulted in decreased proliferation and invasion in CRPC cells (11). Interestingly, TRIM24 could function as transcriptional regulator of both *PIK3CA* and *EGFR* genes in prostate cancer, and that PIK3CA and EGFR had synergetic roles in activation of the PI3K/AKT pathway in prostate cancer and other types of tumors (27, 28). Importantly, we previously proved that both EGFR and PIK3CA are downstream targets of Linc00963 in CRPC (8). Thus, there could be a potential regulation mechanism between Linc00963 and TRIM24. In the current study, we further found TRIM24 was positively correlated with Linc00963 in prostate cancer, and was upregulated by Linc00963 in CRPC. More importantly, we also identified TRIM24 was the downstream target and functional mediator of Linc00963 in CRPC cells.

miR-655 has been identified as tumor suppressive miRNA in ovarian cancer (29), retinoblastoma (30), hepatocellular carcinoma (31), and esophageal squamous cell carcinoma (ESCC) (32). Specifically, miR-655 was markedly decreased in ovarian cancer tissues, and enhancement of miR-655 levels could suppress cell proliferation and invasive ability of SKOV3 cells by directly targeting RAB1A (33). Furthermore, miR-655 expression was lower in NSCLC cell lines than in normal lung fibroblasts. Enhancement of miR-655 levels inhibited the migratory and invasive ability of NSCLC cells by directly suppressing PTTG1 expression (34). Some established oncogenes are also direct targets of miR-655 in other types of cancers, such as Prrx1 in breast cancer (35), ZEB1 and TGFBR2 in ESCC (36), ADAM10 in hepatocellular carcinoma (31), and PAX6 in retinoblastoma (30). However, the functions and targets of miR-655 in CRPC were not identified previously. Here, we provide evidence that miR-655 upregulation decreased the colony forming ability and proliferation of CRPC cells. Functionally, TRIM24 was identified as the direct target of miR-655 in CRPC cells. These results indicate that miR-655 inhibits CRPC proliferation by directly targeting TRIM24.

LncRNAs participate in transcriptional and post-transcriptional regulation, and are involved in the regulation of protein translation through interacting with RNA binding proteins or functioning as ceRNA (37–39). The biological functions of LncRNAs are determined by their subcellular location. If LncRNAs are located in the nucleus, they can activate downstream oncogenes or tumor suppressor genes by interacting with RNA binding protein (40, 41). However, if LncRNAs are located in the cytoplasm, they usually function as ceRNA to upregulate the miRNA target molecules (42, 43). Our study showed that Linc00963 was distributed both in the nucleus and cytoplasm of CRPC cells, indicating that Linc00963 could either interact with RNA binding proteins or function as ceRNA. We noticed that silencing the expression of miR-655 recapitulated effects similar to those observed following enhancement of Linc00963 expression with respect to colony formation and proliferation of CRPC cells. More importantly, TRIM24 was found to be the downstream target of both Linc00963 and miR-655, suggesting that Linc00963 may function as ceRNA to upregulate TRIM24 expression by sponging miR-655. As expected, both luciferase assays and RNA pull-down assays determined that Linc00963 could directly bind miR-655. Through rescue assays, we further confirmed that the Linc00963/miR-655/TRIM24 axis exerted its oncogenic role by promoting cell proliferation and colony formation. We also noticed miR-4731-5p, miR-511-3p, miR-542-3p, miR-1266-3p, miR-532-3p, and miR-10a-5p were identified as the binding miRNAs of Linc00963 in 293T cells by searching the online bioinformatics databases DIANA tools and Starbase (9). But, as we think there are hundreds candidate Linc00963 binding miRNAs, it is very hard to find the candidate miRNAs mediate the regulatory relationship between Linc00963 and target mRNA. Thus, we identified miR-655 as the binding miRNA of Linc00963 by using inverse thinking. We identified the miRNA which regulated the TRIM24 expression by searched for the literatures. Then, we investigated whether the expression of these candidate miRNAs which could inhibit TRIM24 expression directly were suppressed by Linc00963. The different methods should account for the discrepancy of binding miRNAs of Linc00963 in prostate cancer. However, we think these should be other binding miRNAs to link Linc00963 and other oncogenes in prostate cancer.

In conclusion, we revealed that TRIM24 is the downstream target of both Linc00963 and miR-655. Moreover, TRIM24 expression was upregulated by Linc00963 and downregulated by miR-655 in CRPC cells. Finally, we elucidated that Linc00963 promoted cell proliferation and tumor growth of CRPC cells *in vitro* and *in vivo* by sequestering miR-655 and then upregulating TRIM24 expression. Thus, our study contributes to reveal the regulatory mechanism of Linc00963 and TRIM24 in CRPC and may provide novel biomarkers for diagnosis and therapeutic targets for CRPC in future.

## Data Availability Statement

The original contributions presented in the study are included in the article/supplementary material. Further inquiries can be directed to the corresponding author.

## Ethics Statement

The animal study was reviewed and approved by the Animal Care and Use Committee of Xi’an Jiaotong University.

## Author Contributions

MB, SS, CH, MW, PY, YD, and XM conducted the research. SH designed the study. JM contributed the essential reagents or tools. MB and SH wrote the paper. All authors contributed to the article and approved the submitted version.

## Funding

This work was supported by the Innovation Capacity Support Plan of Shaanxi Province (No. 2018TD-002).

## Conflict of Interest

The authors declare that the research was conducted in the absence of any commercial or financial relationships that could be construed as a potential conflict of interest.
